# Supplementary figures and images for: PKR-mediated stress response enhances dengue and Zika virus replication
Source: mBio. 2023 Sep 21;14(5):e00934-23. doi: 10.1128/mbio.00934-23 (PMC10653888; doi:10.1128/mbio.00934-23)

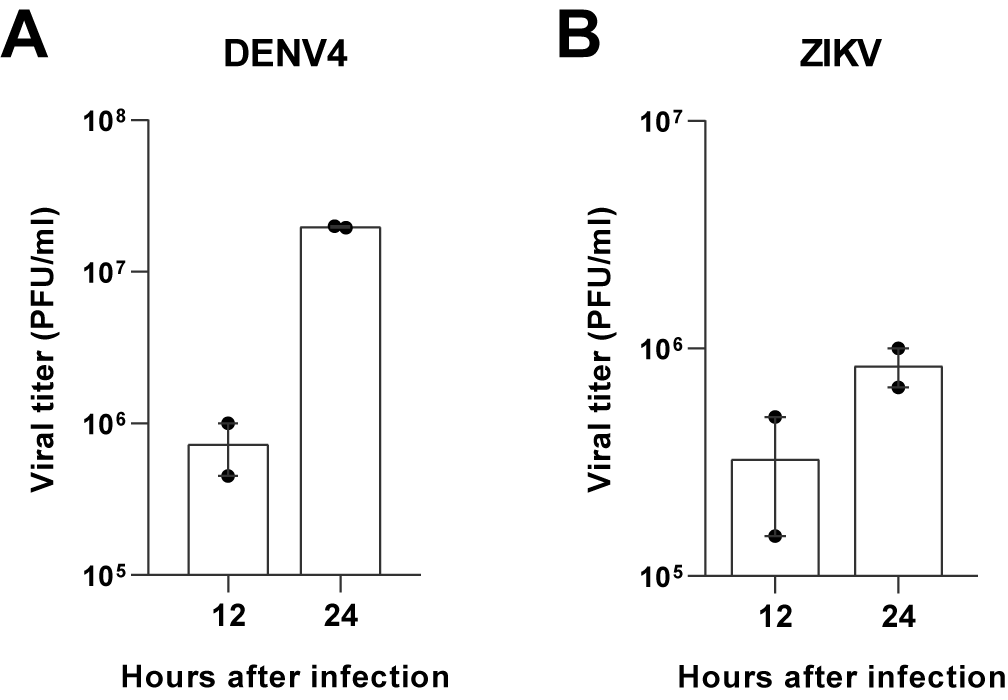

Supplement: Figure S1 — Viral titration in VERO cells of supernatant from A549 WT cells infected with DENV4 or ZIKV. [file mbio.00934-23-s0001.tif]

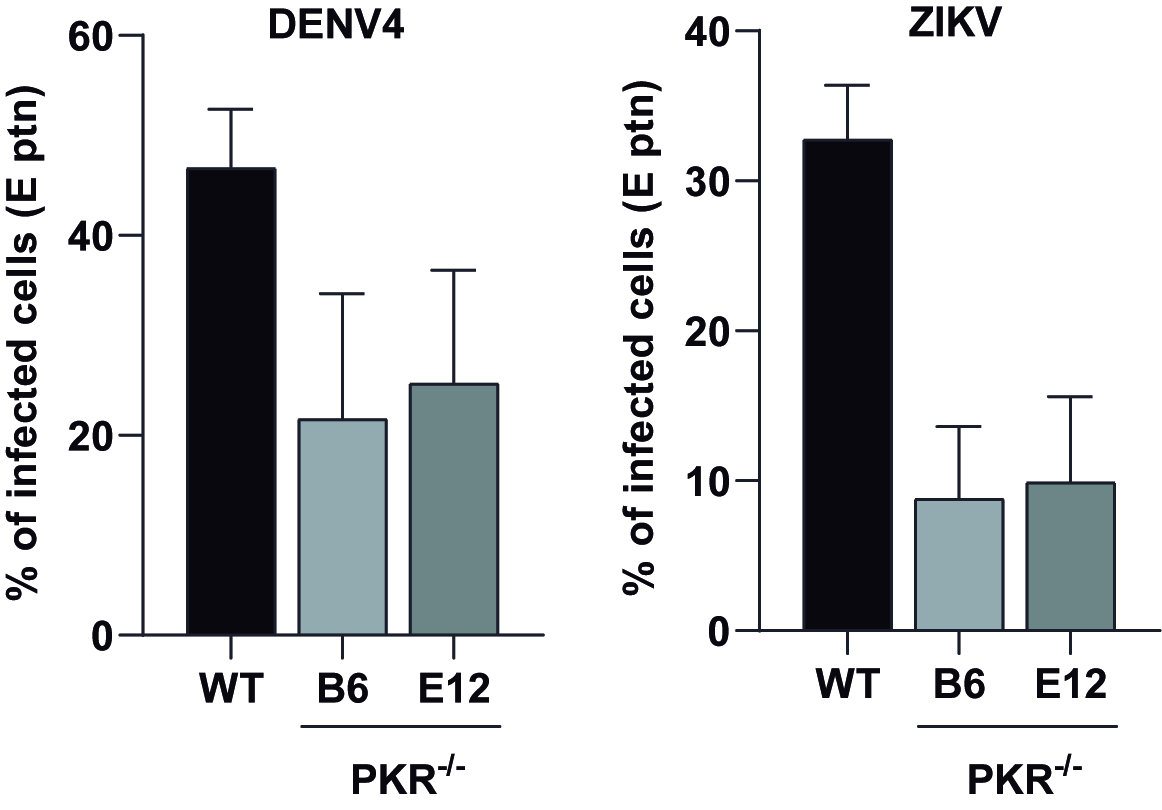

Supplement: Figure S2 — Flow cytometry analysis for quantification of cells expressing p-eIF2α. [file mbio.00934-23-s0002.tif]

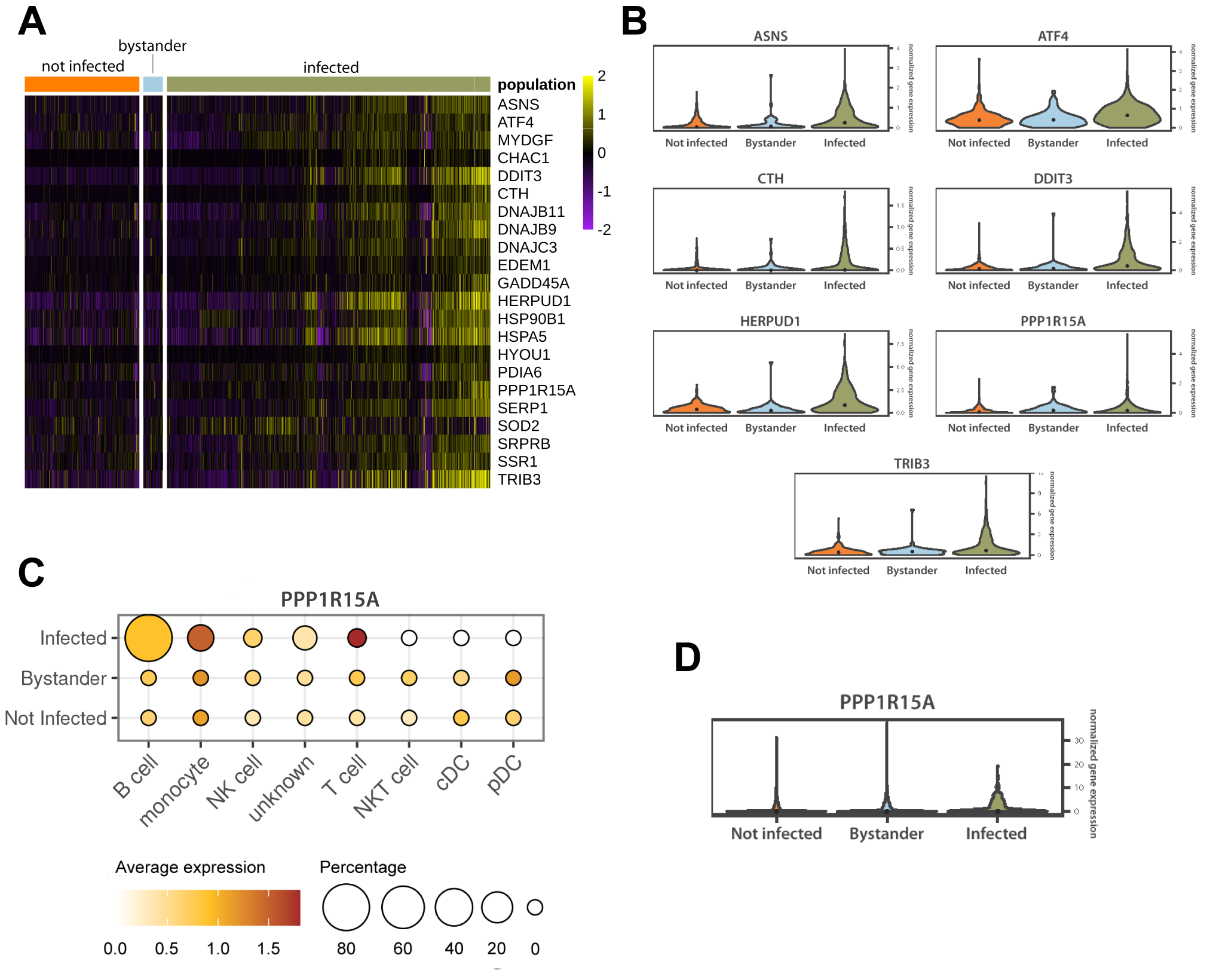

Supplement: Figure S3 — p-eIF2α-downstream genes are upregulated in DENV-infected Huh7 cells and PBMCs. [file mbio.00934-23-s0003.tif]
