# Supplementary material for: PKR-mediated stress response enhances dengue and Zika virus replication
Source: mBio. 2023 Sep 21;14(5):e00934-23. doi: 10.1128/mbio.00934-23 (PMC10653888; doi:10.1128/mbio.00934-23)
Supplement: Legends — for Fig. S1 to S3. [file mbio.00934-23-s0004.docx]

**Supplementary figure legends**

**Supplementary Figure 1** – Viral titration in VERO cells of supernatant from A549 WT cells infected with (A) DENV4 (MOI 2) or (B) ZIKV (MOI 3) harvested at 12 and 24 h p.i. for analysis. PFU: plate forming units; FFU: focus forming units. In all charts, points represent the means ± SEM from three independent experiments.

**Supplementary Figure 2** – A549 WT or PKR-/- (clones B6 or E12) cells infected with DENV4 (MOI 2) or ZIKV (MOI 3) and harvested at 24 hours post-infection. for analysis. Flow cytometry analysis for quantification of cells expressing p‒eIF2α within the total. Bars represent the means ± SEM from two (ZIKV) or three (DENV4) independent experiments.

**Supplementary Figure 3 – p‒eIF2α-downstream genes are upregulated in DENV-infected Huh7 cells and PBMCs.** (A) Dataset from [(55)](https://paperpile.com/c/XzvuFM/1rOHQ), of DENV-infected Huh7 cells, analysed by single-cell RNA-seq. Heatmap representing expression variability of stress-related genes in uninfected, bystander and infected cells; and (B) violin plot representing the expression of p-eIF2α- and ATF4-downstream genes in uninfected, bystander and infected cell populations. (C) Dataset from [(61)](https://paperpile.com/c/XzvuFM/2SqFS), of PBMCs from patients infected with DENV, analysed by single-cell RNA-seq. Dot plot representing the expression of PPP1R15A/GADD34 in different cell population and infection conditions; and (D) violin plot representing the expression of PPP1R15A/GADD34 the whole PBMC population under uninfected, bystander and infected conditions.
